# Endoscopic Band Ligation in Blue Rubber Bleb Nevus Syndrome: A Report of Two Children

**DOI:** 10.1097/PG9.0000000000000344

**Published:** 2023-08-16

**Authors:** Arghya Samanta, Ujjal Poddar, Moinak Sen Sarma, Anshu Srivastava, Surender Kumar Yachha, Samir Mohindra

**Affiliations:** From the Departments of * Pediatric Gastroenterology; † Gastroentrology, Sanjay Gandhi Post Graduate Institute of Medical Sciences, Lucknow, India.

## Abstract

Blue rubber bleb nevus syndrome (BRBNS) is a rare congenital disorder presenting with multifocal venous malformations of the skin, soft tissues, and gastrointestinal (GI) tract. Patients usually present with chronic anemia resulting from occult GI bleeding and sometimes with massive GI bleeding. We report 2 children with blue rubber bleb nevus syndrome with GI bleeding: 1 with obscure GI bleeding and the other with overt GI bleeding. In both cases, the presence of cutaneous lesions provided useful clues toward diagnosis. Colonoscopy and upper GI endoscopy revealed bluish polypoidal lesions in the GI tract. Capsule endoscopy helped in disease mapping. Both of them were successfully treated with endoscopic band ligation and nonselective beta-blockers, which resulted in an improvement in their hemoglobin levels. Our cases highlight the successful use of endoscopic band ligation of GI lesions as a therapeutic modality. It is important for gastroenterologists to be aware of this rare condition for current diagnosis.

## BACKGROUND

Blue rubber bleb nevus syndrome (BRBNS) is a rare congenital disorder characterized by multifocal venous malformations of the skin and other internal organs (1). Besides the skin, the gastrointestinal (GI) tract is the most commonly involved organ. Patients usually present with refractory iron deficiency anemia resulting from GI bleeding (occult or overt) (2). We report 2 children with BRBNS with GI bleeding who were successfully treated with endoscopic band ligation (EBL) and propranolol.

## CASE PRESENTATION

### Case 1

**FIGURE 1.**
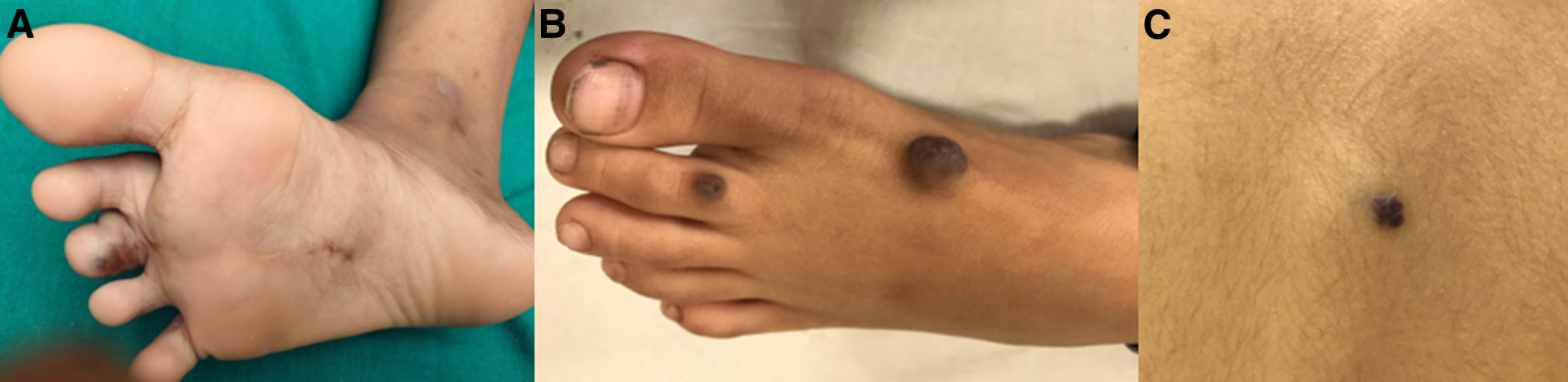
Multiple bluish nodules on the plantar aspect of the right 3rd toe and just above the ankle joint of the first child (A); Similar lesions on the dorsal surface of the left second toe and medial aspect of the dorsum of left foot (B) and posterior trunk (C) of the second child.

An 11-year-old boy presented with recurrent episodes of easy fatiguability and paleness of the body for the past 4 years, which responded to oral iron therapy but recurred once iron was stopped. There was no history of hematemesis, melena, or hematochezia. There was no known family history of vascular disorders. He had a progressively increasing bluish nodule on the right second toe from birth and underwent surgical debulking at 7 years of age. Histopathology showed multiple thickened vessels consistent with arterio-venous malformations. Physical examination revealed multiple blue papules (diameter of 2–5 mm) on the plantar aspect of the right 3rd toe and just above the ankle joint (Fig. 1A).

**FIGURE 2.**
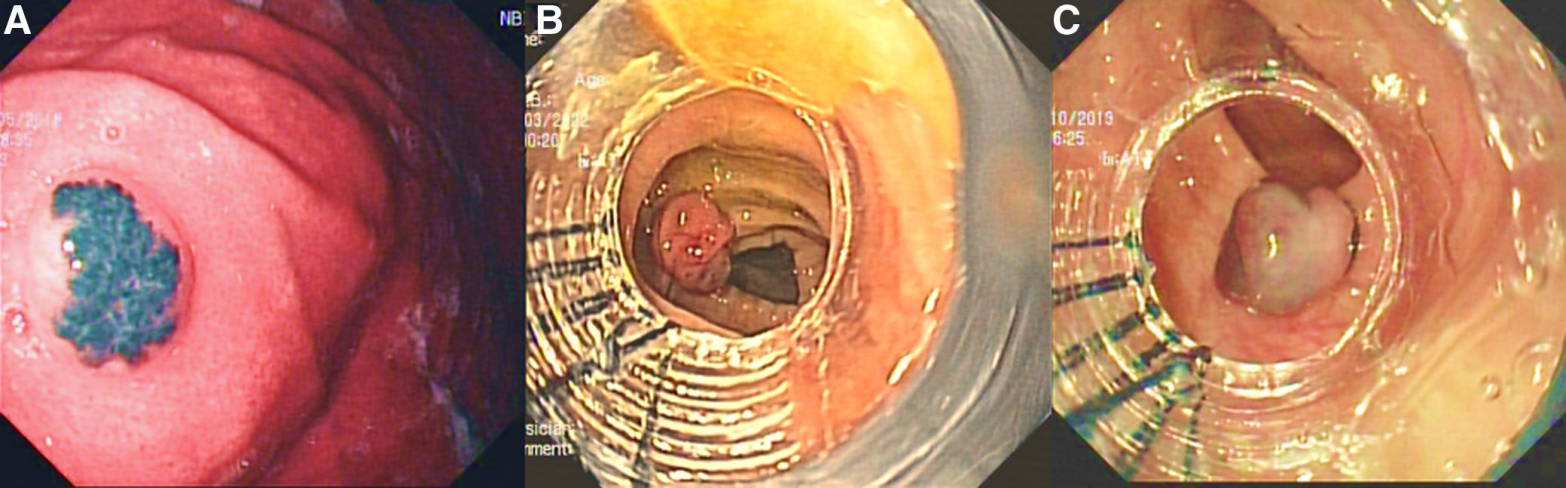
Endoscopic image of a blue bleb-like lesion in the body of the stomach of the first patient (A); Endoscopic band ligation (EBL) of the lesion in the descending colon of the first child, using multiband ligator (B); EBL of colonic lesion of the second child (C).

**FIGURE 3.**
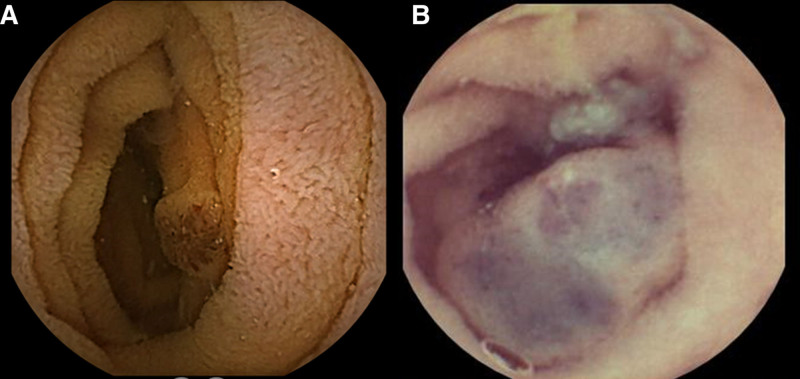
Video capsule endoscopy images showing a blue bleb-like lesion in the small bowel of the first child (A); Similar lesions in the small bowel of the second child (B).

Laboratory findings showed microcytic hypochromic anemia (hemoglobin 6.6 g/dL), reticulocyte count of 1.3%, white blood cell count of 8.0 × 10^9^/L, and platelet count of 189 × 10^9^/L. Serum ferritin level was 9 ng/mL and total iron binding capacity was 569 mg/dL, suggesting iron deficiency anemia. The fecal occult blood test was positive. Colonoscopy revealed 2 bluish polypoidal lesions (approximately 5 mm each) in the sigmoid colon and rectum, while upper gastrointestinal (UGI) endoscopy revealed 2 similar lesions in the gastric body (Fig. 2A). Video capsule endoscopy (VCE) showed 5 similar lesions in the small bowel (Fig. 3A). Deep enteroscopy revealed multiple flat and polypoidal lesions in the distal jejunum and proximal ileum. CT angiography plus enterography did not reveal any vascular lesion in the bowel. The diagnosis of BRBNS was established based on cutaneous lesions and the characteristic endoscopic appearance of the GI lesions. EBL of the lesions in the stomach and colon was performed using a multiband ligator device (Saeed’s multiband ligator; Wilson’s Cook) (Fig. 2B). Although sirolimus was our first choice for maintenance therapy, we opted for propranolol for maintenance therapy because of logistic issues in getting proper monitoring of therapeutic drug levels of sirolimus. At 6 months, the child did not have any further drop in hemoglobin level (hemoglobin of 11.2 g/dL).

### Case 2

A 4-year-old boy presented with a history of intermittent episodes of the passage of blood-mixed stools and occasional melena once every 15–20 days for the past one year. There was no family history of recurrent skin lesions or GI bleeding. He received multiple blood transfusions at 3–6 monthly intervals for the past 2 years. Additionally, he had a history of swelling over his right little toe since birth, which gradually increased in size (diameter of 20 mm) over time. He underwent debulking surgery for the same at 2 years of age. Physical examination showed multiple blue papules (diameter of 5 mm) on the sole of the right foot, dorsum of the left foot, right thumb, and posterior trunk (Fig. 1B and 1C). Colonoscopy and UGI endoscopy revealed multiple bluish polypoidal lesions in the esophagus, gastric body, rectum, and transverse colon. CT angiography plus enterography did not reveal any vascular malformation; however, an ileo-ileal intussusception was detected, which resolved spontaneously. VCE showed similar lesions in the proximal and mid jejunum (Fig. 2B). EBL was carried out thrice in the next 3 years for endoscopically accessible lesions (Fig. 3C). Similar to the first case, this child was also started on propranolol as maintenance therapy. At 8 years of follow-up, the child is not having complaints of any further melena or hematochezia. His blood transfusion requirement reduced significantly (once every 1–2 years). Repeat colonoscopy and UGI endoscopy after 1 year did not show any new lesion but after 3 years showed a few small new lesions in the transverse colon.

## DISCUSSION

The first case of BRBNS was reported in 1860 by Gascoyen et al (3) however, the entity was described in detail by Bean et al (4). Its incidence is relatively low, with only 200 cases reported so far in world literature (5). The pathogenesis is not completely understood, but tyrosine kinase is suspected to play a role (6). Sporadic form of BRBNS is caused by somatic mutations in *TEK* *(tyrosine epithelial kinase),* the gene encoding TIE2 (tunica intima endothelial kinase 2), the endothelial cell tyrosine kinase receptor for the angiopoietins, while activating *germline* mutations in *TEK* causes an autosomal dominantly inherited form of BRBNS (7).

BRBNS presents most commonly as GI bleeding. It can be occult or overt GI bleeding (2,3). One of our patients presented with occult GI bleeding, with chronic iron deficiency anemia, while the other had melena and hematochezia. Lesions are most commonly found in the small intestine and distal large bowel; however, they can be seen anywhere in the GI tract, as has been documented in our cases. The lesions are typically discrete mucosal nodules with a central bluish nipple, although flat, macular, or polypoidal lesions can also be seen. The vascular lesions in the GI tract in this condition can be transmural (which are treated by wedge resection) or mucosal and submucosal (which are amenable to endoscopic band ligation). CT angiography plus CT enterography helps to assess the depth of lesions (8). In both of our cases, CT did not reveal any transmural lesion. The small bowel lesions are best seen at deep enteroscopy and video capsule endoscopy. CT is not specific for hemangiomas (8). Rarely, BRBNS can present with an acute abdomen as a result of intussusception, volvulus, or bowel infarction. The second patient had ileo-ileal intussusception.

Cutaneous lesions are usually present at birth or during early childhood and are mostly located on the tongue, face, trunk, and extremities. These lesions can be macular, papular, nodular or pedunculated, reddish or violaceous, multiple, and are mostly asymptomatic. BRBNS can also involve the central nervous system, eyes, oral cavity, thyroid, parotid gland, musculoskeletal system, lungs, kidney, liver, spleen, and bladder (6).

The treatment of GI lesions depends on the extent of intestinal involvement and the severity of bleeding. For GI lesions, medical therapy with propranolol, steroids, and interferon have been used with variable results (6). Sirolimus, an inhibitor of the mammalian target of rapamycin, has been increasingly used in the management of BRBNS with good efficacy (9). Patients with fewer lesions in the accessible parts of the gut may benefit from endotherapy such as EBL, sclerotherapy, or thermal coagulation (10). In life-threatening situations such as massive GI bleeding, intestinal obstruction, etc., surgical excision may be helpful (11). The use of EBL in GI lesions has been reported scarcely (12,13). In the single case report, Grammatopoulos et al (12) described using EBL and endoloop successfully for jejunal and colonic lesions in a 20-year-old girl, while Guo W et al (13) reported another case of a 22-year-old girl treated with EBL and who did not have a recurrence of GI lesions, assessed endoscopically over the next 3.5 years of follow-up. To the best of our knowledge, this is the first time we are reporting the successful use of EBL for GI lesions in BRBNS as a therapeutic option in children. We suggest that endoscopically accessible mucosal or submucosal lesions should be ligated with EBL, and for prevention, nonselective beta-blockers or sirolimus can be used. To assess the long-term effect of endoscopic followed by medical therapy, surveillance endoscopy at 1–2 years intervals for accessible areas and VCE for small bowel may be considered.

## CONCLUSIONS

Cutaneous vascular lesions can provide useful clues toward the diagnosis of BRBNS. Early diagnosis is crucial to prevent GI bleeding by endoscopic band ligation and starting prophylactic beta-blockers which can be a viable medical treatment for BRBNS.

## ACKNOWLEDGMENTS

All attempts were exhausted in trying to contact the parents/guardians for the purpose of attaining informed consent from the parents or guardians to publish this report for both patients 1 and 2.

## References

[1] Heycock JB Dickinson PH. Haemangiomata of the intestine. Br Med J. 1951;1:620–621. 14821492 10.1136/bmj.1.4707.620 PMC2068552

[2] Narumiya T Nagai Y Kashiwagi H. Endoscopic sclerotherapy for esophageal hemangioma. Gastrointest Endosc. 2000;52:285–287. 10922114 10.1067/mge.2000.107215

[3] Gascoyen GG. Case of nevus involving the parotid gland and causing death from suffocation: nevi of the viscera. Trans Pathol Soc Lond. 1860;11:267.

[4] Bean WB. “Blue rubber bleb nevi of the skin and gastrointestinal tract”. In: Bean WB, editor. Springfield, IL, United States. Vascular Spiders and Related Lesions of the Skin; 1958;5:178–185.

[5] Oksuzoglu BC Oksuzoglu G Cakir U. Blue rubber bleb nevus syndrome. Am J Gastroenterol. 1996;91:780–782. 8677949

[6] Jin XL Wang ZH Xiao XB. Blue rubber bleb nevus syndrome: a case report and literature review. World J Gastroenterol. 2014;20:17254–17259. 25493043 10.3748/wjg.v20.i45.17254 PMC4258599

[7] Wouters V Limaye N Uebelhoer M. Hereditary cutaneomucosal venous malformations are caused by TIE2 mutations with widely variable hyper-phosphorylating effects. Eur J Hum Genet. 2010;18:414–420. 19888299 10.1038/ejhg.2009.193 PMC2841708

[8] Certo M Lopes L Ramada J. Blue rubber bleb nevus syndrome: manifestations at computed tomography. Acta Radiol. 2007;48:962–966. 17957509 10.1080/02841850701477702

[9] Karastaneva A Gasparella P Tschauner S. Indications and limitations of sirolimus in the treatment of vascular anomalies-insights from a retrospective case series. Front Pediatr. 2022;10:857436. 35676905 10.3389/fped.2022.857436 PMC9168223

[10] Aoyama T Fukumoto A Shigita K. Successful endoscopic sclerotherapy using polidocanol for small bowel hemangioma. Intern Med. 2020;59:1727–1730. 32238724 10.2169/internalmedicine.4327-19 PMC7434551

[11] Shahed M Hagenmuller F Rosch TH. A 19-year-old female with blue rubber bleb nevus syndrome: endoscopic laser photocoagulation and surgical resection of gastrointestinal angiomata. Endoscopy. 1990;22:54–56. 2307133 10.1055/s-2007-1012789

[12] Grammatopoulos A Petraki K Katsoras G. Combined use of band ligation and detachable snares(endoloop) in a patient with blue rubber bleb nevus syndrome. Ann Gastroenterol. 2013;26:264–266. 24714239 PMC3959436

[13] Guo W Peng Z Tang X. Endoscopic management of blue rubber bleb nevus syndrome: a case report. Exp Ther Med. 2013;6:1159–1162. 24223638 10.3892/etm.2013.1303 PMC3820848

